# Exercise responses to perceptually regulated high intensity interval exercise with continuous and intermittent hypoxia in inactive overweight individuals

**DOI:** 10.1113/EP092338

**Published:** 2025-02-12

**Authors:** Jacky Soo, Paul Goods, Olivier Girard, Louise Deldicque, Nathan G. Lawler, Timothy J. Fairchild

**Affiliations:** ^1^ Discipline of Exercise Science, School of Allied Health Murdoch University Perth Australia; ^2^ The Centre for Healthy Aging, Health Futures Institute Murdoch University Perth Australia; ^3^ School of Human Sciences (Exercise and Sport Science) The University of Western Australia Crawley Western Australia Australia; ^4^ Institute of Neuroscience UCLouvain Louvain‐la‐Neuve Belgium; ^5^ Australian National Phenome Centre, Health Futures Institute Murdoch University Perth Australia; ^6^ The Centre for Molecular Medicine and Innovative Therapeutics, Health Futures Institute Murdoch University Perth Australia

**Keywords:** environmental stress, hypoxic conditioning, internal load, muscle oxygenation, perceptual responses

## Abstract

To investigate the acute effects of hypoxia applied during discrete work and recovery phases of a perceptually regulated, high‐intensity interval exercise (HIIE) on external and internal loads in inactive overweight individuals. On separate days, 18 inactive overweight (28.7 ± 3.3 kg m^−2^; 31 ± 8 years) men and women completed a cycling HIIE protocol (6 × 1 min intervals with 4 min active recovery, maintaining a perceived rating of exertion of 16 and 10 during work and recovery, respectively, on the 6–20 Borg scale) in randomized conditions: normoxia (NN), normobaric hypoxia (inspired O_2_ fraction ∼0.14) during both work and recovery (HH), hypoxia during recovery (NH) and hypoxia during work only (HN). Markers of external (relative mean power output, MPO) and internal load (blood lactate concentration, heart rate and tissue saturation index (TSI)) were measured. MPO was lower in HH compared to NN, NH and HN (all *P *< 0.001), with HN also being lower than NN (*P *< 0.001) and NH (*P *< 0.023). Heart rate was higher in HN than NN, HH and NH (all *P *< 0.001). Blood lactate response was higher in NN than HH (*P *= 0.003) and NH (*P *= 0.008). Changes in the TSI area above the curve were greater in HN relative to NN, HH and NH (all *P *< 0.001). Hypoxia applied intermittently during the work or recovery phases may mitigate the declines in mechanical output observed when exercise is performed in continuous hypoxia, although hypoxia implemented during the work phase resulted in elevated heart rate and lactate response. Specifically, exercise performance largely comparable to that in normoxia can be achieved when hypoxia is implemented exclusively during recovery.

## Abstract (highlights)

- **What is the central question of this study?**
  How does intermittent hypoxia applied during different phases, (i.e. work, recovery and both) of a perceptually regulated high‐intensity interval exercise influence exercise performance, metabolic and perceptual responses in inactive overweight individuals?
- **What is the main finding and its importance?**
  Intermittent hypoxia, implemented during either the work or recovery phase of HIIE attenuated significant declines in mechanical output associated with continuous hypoxia, but did not alter acute metabolic responses beyond that exercise in normoxia. The application of intermittent hypoxic exposure could be an effective method to harness the benefits of decreased oxygen saturation without compromising absolute exercise intensity, thereby enhancing the acute effects of exercise in this population.

## 1 INTRODUCTION

Physical inactivity has contributed to the rising global prevalence of obesity and comorbid conditions in recent decades (Ladabaum et al., 2014). A common barrier to increasing physical activity is the ‘lack of time’ (Trost et al., 2002). Accordingly, exercise prescription has shifted toward shorter duration, higher‐intensity exercise (Bird & Hawley, 2012). High‐intensity interval exercise (HIIE), defined as brief (≤4 min) intermittent bouts of high‐intensity efforts, eliciting greater than 80% of peak heart rate, with shorts period of recovery (Callahan et al., 2021), has emerged as an effective intervention that induces positive health outcomes (Coates et al., 2023; Maturana et al., 2021).

Two important components in designing an effective HIIE protocol are exercise intensity and duration, which ultimately influence acute physiological responses and long‐term adaptations. In this context, exercise intensity is typically prescribed at a predetermined level, for instance, relative to an individual's maximal aerobic power or heart rate determined during a graded exercise test (Daussin et al., 2008; Little et al., 2011). However, findings have demonstrated that when the intensity of interval exercise is prescribed relative to ${\dot{V}}_{O_{2}\max}$, heterogeneity in exercise tolerance is observed such that individuals may not be able to complete the exercise session (Bossi et al., 2024; Meyler et al., 2023); this may, in turn, affect an individual's self‐efficacy, and consequently long term adherence to exercise.

Alternatively, during HIIE, exercise intensity can be guided by an individual's exercise‐related sensations (rating of perceived exertion; RPE), experienced during both the work and recovery phases (Seiler & Hetlelid, 2005; Seiler & Sjursen, 2004). In particular, RPE is considered a useful metric that can be used with physiological measures including heart rate (Green et al., 2006) and/or blood lactate (Stoudemire et al., 1996) to regulate exercise intensity, regardless of an individual's fitness level (Seip et al., 1991). Accordingly, perceptually regulated interval exercise may be particularly applicable for physically inactive individuals.

One proposed mechanism explaining the effectiveness of HIIE is the significant acute metabolic perturbations induced by the intensity of exercise during the work phase (Combes et al., 2015). Transient responses including a localized decrease in partial pressure of oxygen within the exercising muscle and increased substrate utilization may influence the beneficial longer‐term physiological adaptations (Egan & Sharples, 2023). In this regard, the use of high intensity interval exercise may impart greater therapeutic benefits for glycaemic control (De Groote & Deldicque, 2021; Soo et al., 2023). Intense exercise induces a shift in the pattern of substrate utilization with greater reliance on skeletal muscle glycogen. From a functional standpoint, the observed post‐exercise improvement in insulin sensitivity appears necessary for the replenishment of muscle glycogen (Fell et al., 1982). These responses may also be augmented when exercising in hypoxia. Specifically, a decrease in oxygen saturation leads to increased muscle tissue deoxygenation. Consequently, hypoxic exercise performed at the same absolute intensity as in normoxia increases glucose uptake, muscle glycogen breakdown, and consequently, greater muscle and plasma lactate concentration (Wadley et al., 2006).

Accordingly, HIIE in hypoxia has been suggested as a strategy to enhance these longer‐term adaptations in athletes (Faiss et al., 2013). More recently, HIIE in hypoxia has been shown to be an effective strategy to improve cardiorespiratory fitness and cardiometabolic health in individuals with overweight (Camacho‐Cardenosa et al., 2018; Kong et al., 2017). However, a potential drawback with conventional HIIE in hypoxia is a decline in absolute exercise intensity relative to HIIE in normoxia (Billaut & Buchheit, 2013). The resultant decline in exercise intensity may blunt the exercise‐associated physiological and metabolic responses. Indeed, the decrease in absolute exercise intensity in individuals with overweight or obesity was postulated to explain the lack of additional cardiometabolic benefits resulting from an 8‐week HIIE training programme in continuous hypoxia, beyond exercise training in normoxia (Ghaith et al., 2022).

A novel method of implementing hypoxia during HIIE involves using intermittent hypoxic exposure, that is, only during work or recovery phase. For example, applying hypoxic exposure *only* during the recovery phase of HIIE may help individuals maintain their exercise intensity during the work bouts, effectively replicating exercise performance in normoxia. Additionally, applying hypoxia during recovery decreases oxygen saturation, possibly activating signallings pathway associated with metabolic benefits (e.g. improved regulation of glucose) (Soo et al., 2023). However, it is possible that the reduction in oxygen saturation during recovery may decrease recovery kinetics (e.g. phosphocreatine resynthesis), reducing exercise performance during the subsequent intervals. Alternatively, hypoxic exposure only during the work bouts may enhance the exercise‐induced metabolic processes (e.g. glycolytic flux, glycogen depletion and lactate production), while the recovery in normoxia may minimize hypoxia‐associated declines in performance. However, it remains unclear whether intermittent hypoxia, applied during either the work or recovery phase of HIIE, can harness the benefits of decreased oxygen saturation without compromising absolute exercise intensity. Considering the low exercise capacity in physically inactive individuals who are overweight (Vaccari et al., 2019), the application of intermittent hypoxic exposure during perceptually regulated HIIE may be an effective method to enhance the acute effects of exercise in this population.

As such, the aim of this study was to investigate the acute effects of intermittent hypoxia during different phases, that is, work, recovery and both, of a perceptually regulated HIIE on exercise performance, metabolic and perceptual responses in inactive overweight individuals. It was hypothesized that, compared to perceptually regulated HIIE in normoxia, sustained mechanical load would be lowest when hypoxia is applied throughout the session. Additionally, intermittent hypoxic exposure, during either work or recovery, would attenuate the decline in mechanical load, with the smallest decline occurring when hypoxia is applied *only* during the recovery period. Finally, we hypothesized that heart rate and blood lactate would be highest when hypoxia is applied only during the intermittent work bouts.

## 2 METHODS

An a priori power analysis revealed 16 participants would be required to detect a medium effect (*f* = 0.25; type I error = 0.05; type II error = 0.95) between the four repeated conditions, assuming four repeated measurements with a correlation between measures of 0.80. We anticipated some (∼10%) dropout or missing data, and therefore 18 inactive (defined as less than two scheduled aerobic exercise sessions and 100 min of moderate to vigorous intensity aerobic exercise per week) and overweight individuals (i.e., body mass index ≥ 25 kg/m^2^) were recruited to participate in this study (Table 1). They comprised 13 males (age: 31 ± 8 years; body mass index: 28.0 ± 3.0 kg/m^2^) and five females (age: 32 ± 10 years; body mass index: 30.5 ± 3.6 kg/m^2^). Participants were excluded if they were smokers, had uncontrolled hypertension (blood pressure greater than 160/90 mmHg), prior history of heart disease, and contraindications to exercise assessed using the *Exercise & Sports Science Australia* (ESSA) pre‐exercise screening tool. Additionally, individuals who were born and raised at >1500 m and/or had travelled to elevations >1000 m within 3 months prior to the study were excluded. Before commencement of the study, participants were informed of the experimental procedures and possible benefits and risks associated with the study, and a written informed consent was obtained prior to participation. This study was approved by the Murdoch University Human Research Ethics Committee (2022/074) and performed in accordance with the *Declaration of Helsinki*, except for registration in a database.

**TABLE 1:** Physical characteristic of participants.

**Participant characteristics** (*n* = 18; 13 males, 5 females)			
	Male	Female	All
Age (years)	30.9 ± 8.5	32.4 ± 9.9	31.3 ± 8.1
Weight (kg)	90.2 ± 11.3	85.8 ± 17.6	89.0 ± 12.8
Height (cm)	179.5 ± 5.8	167.1 ± 10.2	176.1 ± 9.4
BMI (kg/m^2^)	28.0 ± 3.1	30.5 ± 3.6	28.7 ± 3.3
Body fat percentage (%)	26.3 ± 5.5	38.1 ± 6.4	29.6 ± 8.1
Fat free mass (kg)	64.7 ± 6.6	51.5 ± 7.3	61.0 ± 9.0
${\dot{V}}_{O_{2}\mathrm{peak}}$ (ml/kg/min)	33.6 ± 9.6	23.0 ± 4.3	30.7 ± 8.9
${\dot{V}}_{O_{2}\mathrm{peak}}$ (ml/kg FFM/min)	46.6 ± 10.7	37.9 ± 6.2	44.2 ± 10.3
*Ẇ*_peak_ (W)	259 ± 51	181 ± 30	237 ± 58
Relative *Ẇ*_peak_ (W/kg)	2.9 ± 0.6	2.1 ± 0.3	2.7 ± 0.6
Relative *Ẇ*_peak_ (W/kg FFM)	4.0 ± 0.7	3.5 ± 0.3	3.9 ± 0.6

*Note*: Data are means  ± SD. Abbreviations: BMI, body mass index; FFM, fat free mass; ${\dot{V}}_{O_{2}\mathrm{peak}}$, peak oxygen uptake; *Ẇ*_peak_, peak power output.

### 2.1 Experimental design

All participants completed five sessions, which included one familiarization session and four main experimental trials. All experimental sessions were conducted in a randomized, crossover, and counterbalanced design (Latin‐square) separated by 4–7 days and conducted at the same time of day.

During the first visit, participants had their body mass and height measured to determine their body mass index. Body composition was then assessed using dual energy X‐ray absorptiometry (Discovery Series W; Hologic, Mississauga, Canada) to determine body fat percentage. Finally, blood pressure and peak oxygen consumption (${\dot{V}}_{O_{2}\mathrm{peak}}$) were measured. Following completion of the baseline testing, participants performed a familiarization trial to acquaint themselves with the exercise protocol, experimental procedures and equipment to be used during the subsequent experimental sessions.

All participants completed four separate experimental sessions, each involving HIIE under the following conditions: (1) normoxic exercise–normoxic recovery (NN; normoxia throughout the entire session); (2) hypoxic exercise–hypoxic recovery (HH; hypoxia throughout the entire session); (3) normoxic exercise–hypoxic recovery (NH; hypoxia during between‐intervals recovery period only); and (4) hypoxic exercise–normoxic recovery (HN; hypoxia during work intervals only). All aspects of the procedures were identical, except for the application of hypoxic exposure during the work and/or recovery phases. For each experimental session, participants were instructed to avoid vigorous exercise for 24 h, and caffeine for 12 h prior to exercise. Additionally, they were asked to consume their last meal 2–3 h before arriving at the laboratory and to replicate their dietary intake as closely as possible.

### 2.2 Baseline and familiarization session

Participants performed an incremental test to exhaustion on an electromagnetically braked cycle ergometer (Lode Corival, Groningen, The Netherlands) to assess ${\dot{V}}_{O_{2}\mathrm{peak}}$, peak heart rate (HR), and associated peak power output (*Ẇ*_peak_). The test started with 2 min of cycling at 50 W, after which work rate increased by 15 W every min until volitional exhaustion. Pulmonary gas exchanges were measured continuously (Parvomedics Trueone 2400, Sandy, USA), and ${\dot{V}}_{O_{2}\mathrm{peak}}$ was defined as the highest ${\dot{V}}_{O_{2}}$ over a 15 s epoch.

Following the incremental exercise, participants rested for ∼30 min before being familiarized with the actual exercise intervention. Specifically, a ramped cycling test was performed on an electronically braked cycle ergometer (Velotron, Racermate, Seattle, USA) to familiarize participants with the intensity corresponding to RPE of 16 (denoted as between *hard* and *very hard* on the 6–20 Borg scale) and 10 (between *very light* and *light*). This ramp test was adapted from a previous protocol designed to identify treadmill running speed corresponding to a RPE of 16 (Hobbins et al., 2021). The first ramp test started at an intensity of 50 W and increased by 20 W every 30 s until a RPE ≥ 16 was reached. The second ramp test started at 20 W higher than the end intensity of the first test, decreasing by 20 W every 30 s until a RPE ≤ 10 was reached. The RPE was recorded at the end of each 30 s stage. Participants were provided with 1–2 min of rest between each stage to minimize residual fatigue. Finally, they performed 2 × 1 min high‐intensity effort at a RPE of 16 with 4 min of recovery (at a RPE of 10) between efforts. The participants were blinded to the exercise intensity (i.e., workload) throughout all sessions.

### 2.3 Experimental trials

Before each HIIE session, participants performed a standardized warm‐up in normoxia. The warm‐up procedures were as follows: (1) 3 min of continuous cycling (1 min bouts at a RPE of 10, 12 and 14, respectively), (2) 3 min of passive rest, and (3) 2 × 30 s bouts at a RPE of 16, with 2 min of passive rest between each bout. This was followed by 6 min of seated rest on the cycling ergometer (wash‐in period) under normoxic conditions in NN and NH, and hypoxic conditions in HH and HN before the start of the HIIE.

The HIIE protocol consisted of 6 × 1 min work bouts interspersed with 4 min recovery for a total exercise duration of 30 min. Participants were asked to perform the work and recovery bouts at a perceptually regulated intensity corresponding to a RPE of 16 and 10, respectively. An active recovery was used as this may potentially facilitate faster ${\dot{V}}_{O_{2}}$ kinetics (therefore longer time spent at an elevated ${\dot{V}}_{O_{2}}$) (Buchheit & Laursen, 2013) and muscle deoxygenation kinetics (Dupont et al., 2004) during the subsequent work bouts. Consequently, a 4 min recovery duration was chosen to ensure maintenance of high intensity performance (avoiding premature fatigue) during the subsequent bouts. Cadence and gearing were self‐selected by participants to ensure that exercise was performed at the prescribed intensity during each work and recovery bout (i.e., RPE of 16 and 10). Verbal encouragement was provided at regular intervals (every 15 s) during each 1 min bout to ensure that participants maintained the prescribed exercise intensity. All sessions were conducted using the same Velotron cycling ergometer at thermo‐neutral conditions (20°C, 40% relative humidity) in an environmental chamber.

Power output was calculated and sampled at a rate of 1 Hz via an algorithm within the Velotron software. Power output data collected during the exercise were subsequently downloaded for offline analysis to determine the mean power output (MPO) for each work and recovery bout. MPO was normalized by total body mass and *Ẇ*_peak_ (determined during the incremental test) for subsequent analysis. Additionally, fatigue during each session was calculated from MPO using the formula (Glaister et al., 2004):

$$\mathrm{Fatigue}\;=(100\;\times \;e^{\left[slope/100\right]}\;-\;100),$$

where slope = (The slope of the line of best fit for the natural log of MPO × 100) × (number of intervals – 1).

### 2.4 Hypoxia exposure

Participants wore a facemask that was connected to a portable hypoxic generator (AltiTrainer; Smtec AG, Kleinandelfingen, Switzerland) via a corrugated plastic tubing during all experimental sessions. A two‐way valve that allows for switching between normoxic (fraction of inspired oxygen ($F_{iO_{2}}$) ∼0.21) and hypoxic air ($F_{iO_{2}}$ ∼0.14, corresponding to a simulated altitude of ∼3000 m) was connected to the corrugated tubing. The choice of a $F_{iO_{2}}$ of 0.14 was based on previous studies showing its safety and efficacy for inducing adequate metabolic stress to alter glucose metabolism in overweight individuals (De Groote et al., 2021; Mackenzie et al., 2011). For blinding purposes, the two‐way valve system was positioned out of the participants’ view and the hypoxic generator was positioned outside of the environmental chamber.

### 2.5 Exercise responses

Heart rate (Garmin HRM‐Dual Garmin Ltd, Kansas, USA), peripheral oxygen saturation ($S_{pO_{2}}$; WristOx2 3150; Nonin Medical Inc., Amsterdam, the Netherlands), and perceptual responses (difficulty breathing and limb discomfort, assessed via a modified Borg CR10 scale) were recorded ∼10 s after each 1 min bout. Capillary blood samples were taken from the fingertip and analysed for blood lactate levels with a lactate analyser (Lactate Plus, Nova Biomedical, Waltham, MA, USA) at baseline (before any exercise), 1 min before the start of the HIIE protocol, ∼15 s after each work bout, and ∼45 s before the start of the next 1 min bout. Lactate response after each 1 min bout was normalized by MPO to examine the relationship between power output and lactate production.

Changes in oxygenation in the gastrocnemius lateralis muscle were measured using near‐infrared spectroscopy (NIRS). Specifically, a portable NIRS continuous wave photometer (Portamon, Artinis Medical Systems, BV, Elst, The Netherlands) was positioned on the right leg, at approximately one‐third of the distance between the head of the fibula and the heel. The probe was secured using 3M Transpore surgical tape, and a dark elastic bandage was wrapped around the probe to prevent ambient light contamination. The skin overlying the muscle region was shaved, and adipose tissue thickness was measured using a skinfold caliper before placing the probe. All procedures were performed by the same experimenter to ensure consistency between sessions.

The sampling frequency was set at 10 Hz and data were exported at 1 Hz for offline analysis. All NIRS data were independently reviewed by two researchers to assess data quality, and a third reviewer was consulted in case of disagreement. NIRS data for four participants were excluded from the analysis following review (due to significant artefacts in three or more sessions out of four). In total, NIRS data for 12, 11, 10 and 13 participants were deemed suitable for subsequent analysis in NN, HH, NH and HN, respectively.

**FIGURE 1:**
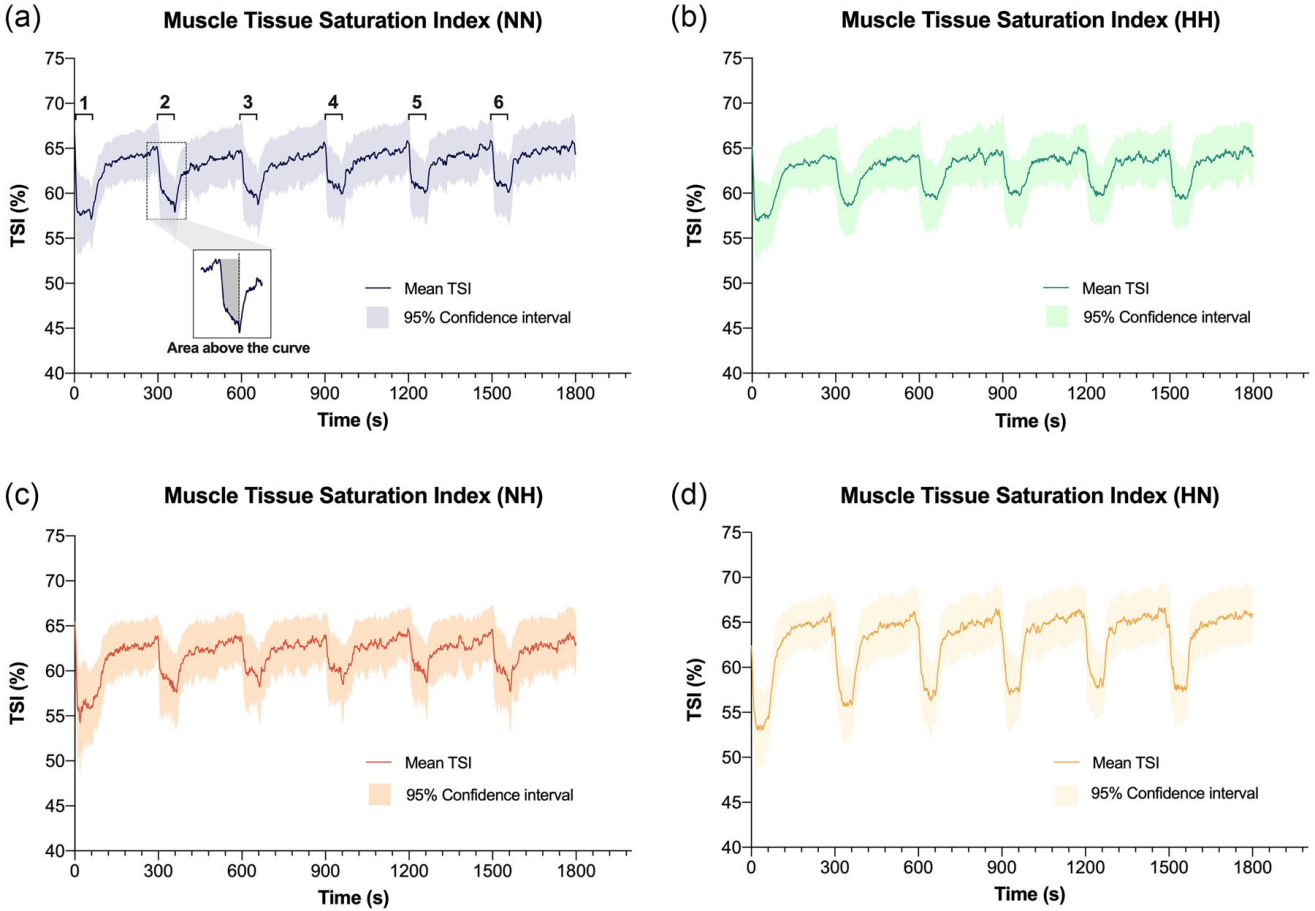
Mean muscle TSI (with 95% confidence interval band) during the HIIE under normoxic exercise–normoxic recovery (NN; blue; a), hypoxic exercise–hypoxic recovery (HH; green; b), normoxic exercise–hypoxic recovery (NH; orange; c), and hypoxic recovery–normoxic recovery (HN; yellow; d). Start and end of intervals 1–6 are indicated in panel (a). Representative area above the curve determination for one interval is presented in panel (a). HH, continuous hypoxia; HIIE, high‐intensity interval exercise; HN, exercise in hypoxia; NH, recovery in hypoxia; NN, exercise under normoxia; TSI, tissue saturation index.

To determine changes in muscle oxygenation during exercise, the tissue saturation index (TSI) area above the curve (Soares et al., 2017) for each 1 min work interval was calculated. Specifically, this involved calculating the total area in the TSI signal from the highest value recorded during the 30 min exercise to the end of each 1 min exercise bout (Figure 1a).

### 2.6 Statistical analysis

Mechanical output (relative MPO), physiological (HR and $S_{pO_{2}}$) and perceptual (limb discomfort and breathing difficulty) responses, and gastrocnemius lateralis muscle oxygenation were analysed using linear mixed method modelling between condition (NN, HH, NH and HN) and time (intervals 1–6). Condition and time were included as fixed effects and participants were treated as random effects. Similarly, differences in blood lactate response were analysed using linear mixed method modelling, with fixed effect for condition (NN, HH, NH and HN) and time (0 min, pre and post each interval, and end‐exercise) and participants as random effects. A two‐way repeated‐measures analysis of variance (Trials [experimental sessions 1, 2, 3, 4] × Time [intervals 1–6]) was used to compare differences in mechanical output to determine the effects of trial sequence on exercise performance. Significant main effects or interactions were analysed using Fisher's LSD *post hoc* analysis. Effects size estimates, Hedge's *g*, were also calculated and interpreted as small (*g *= 0.2), moderate (*g *= 0.5) or large (*g *= 0.8) (Cohen, 1988). Statistical analyses were conducted using SPSS Statistics (version 24; IBM Corp., Armonk, NY) and variables were deemed significant when *P* ≤ 0.05. Significant differences between conditions are indicated by symbols and differences between intervals are denoted by letters. Data are reported as mean ± standard deviation (SD) unless otherwise stated.

## 3 RESULTS

### 3.1 Exercise performance

**FIGURE 2:**
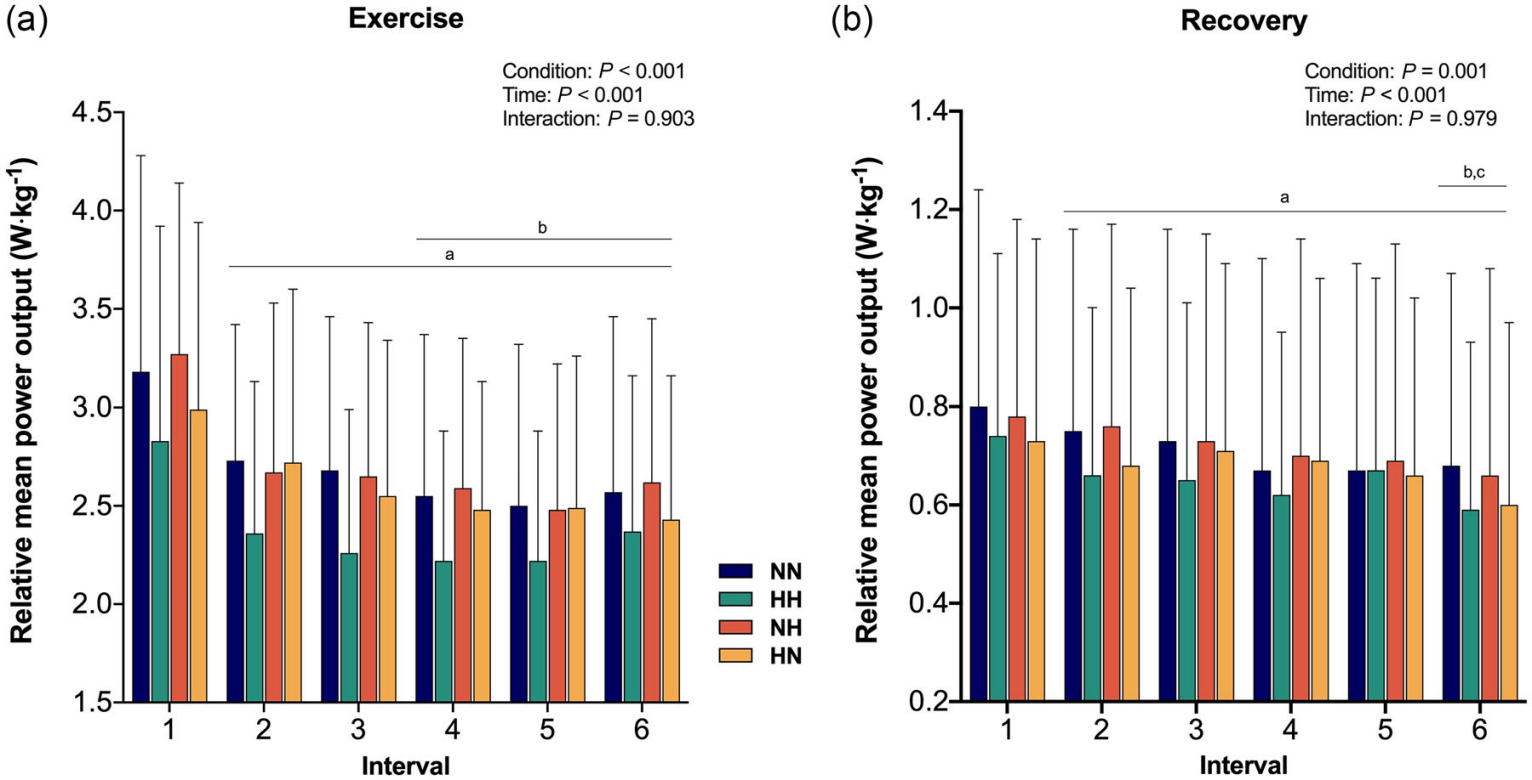
Relative MPO of exercise (a) and recovery (b) during the HIIE under normoxic exercise–normoxic recovery (NN; blue), hypoxic exercise–hypoxic recovery (HH; green), normoxic exercise–hypoxic recovery (NH; orange), and hypoxic recovery–normoxic recovery (HN; yellow). ^a^Significant difference from interval 1 (*P* < 0.05); ^b^significant difference from interval 2 (*P* < 0.05); ^c^significant difference from interval 3 (*P* < 0.05). HH, continuous hypoxia; HIIE, high‐intensity interval exercise; HN, exercise in hypoxia; MPO, mean power output; NH, recovery in hypoxia; NN, exercise under normoxia.

Changes in relative MPO (normalized by total body mass) displayed a main effect of condition (*P* < 0.001) and time (*P* < 0.001). Specifically, relative MPO was lower in HH (all *P* < 0.001, *g* = 0.28–0.41) compared to NN (mean between‐group difference and 95% confidence interval: 0.32 [0.23–0.42] W/kg), NH (0.34 [0.24–0.43] W/kg) and HN (0.21 [0.12–0.31] W/kg; Figure 2a). Additionally, MPO was lower in HN (all *P* < 0.023, *g* = 0.12–0.13) compared to NN (0.11 [0.02–0.21] W/kg) and NH (0.12 [0.03–0.22] W/kg). When expressed as a percentage of the *Ẇ*_peak_, the power output during the 1 min bouts (pooled average from intervals 1–6) were 99.1 ± 25.1%, 88.3 ± 23.1%, 100.2 ± 21.7% and 97.7 ± 22.6% for NN, HH, NH and HN, respectively. Fatigue index did not differ between conditions (17.7 ± 12.3%, 11.2 ± 17.8%, 17.4 ± 12.1% and 11.5 ± 17.7% for NN, HH, NH and HN, respectively; *P* = 0.30). Changes in mechanical output during exercise were not affected by the trial sequence (main effect of trial, *P* = 0.68).

### 3.2 Physiological and metabolic responses

**FIGURE 3:**
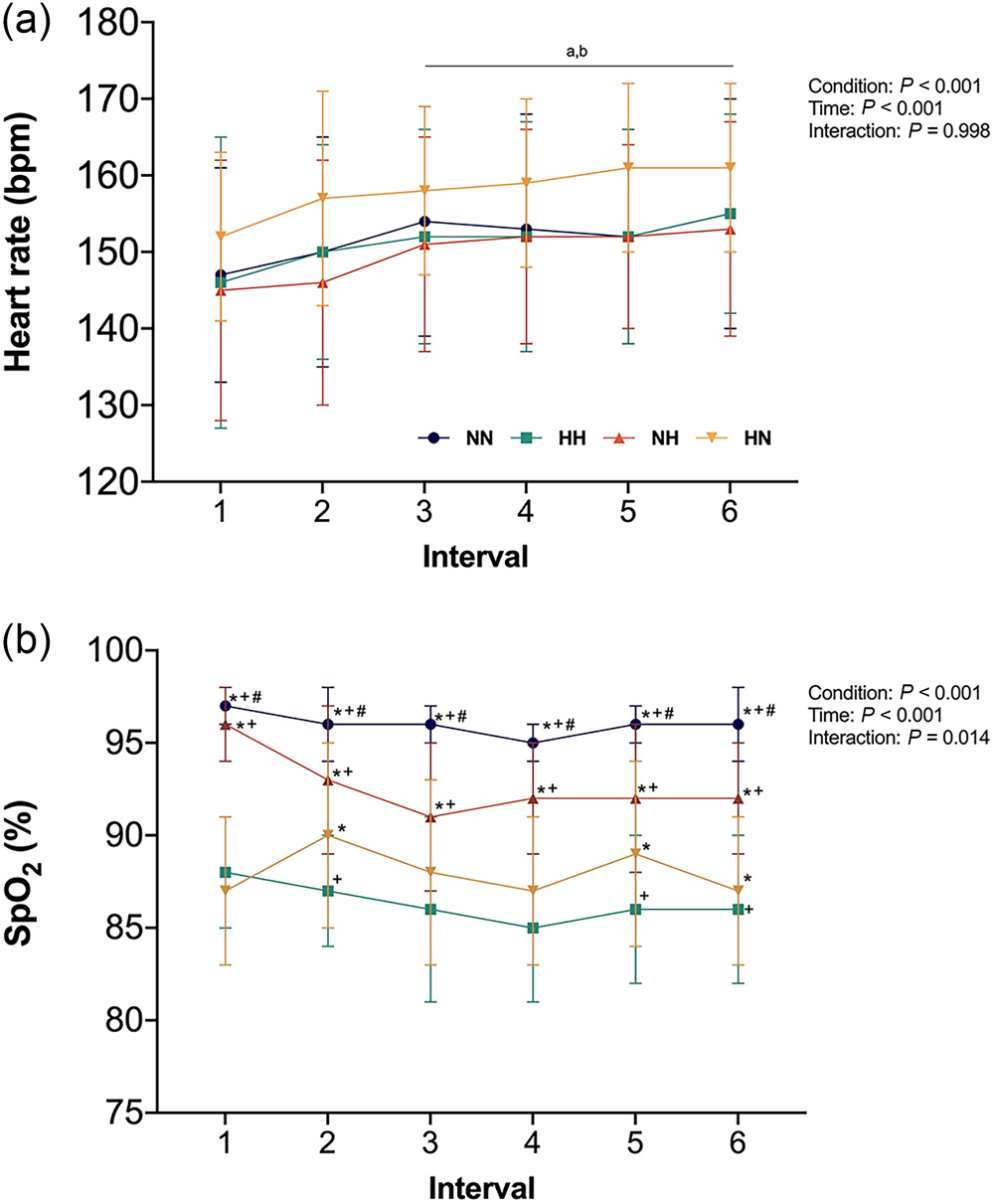
Heart rate (HR; a) and arterial oxygen saturation ($S_{pO_{2}}$; b) during each interval of the HIIE under normoxic exercise–normoxic recovery (NN; blue), hypoxic exercise–hypoxic recovery (HH; green), normoxic exercise–hypoxic recovery (NH; orange), and hypoxic recovery–normoxic recovery (HN; yellow). ^a^Significant difference from interval 1 (*P* < 0.05); ^b^significant difference from interval 2 (*P* < 0.05); ^∗^significant difference from HH (*P* < 0.05); ^+^significant difference from HN (*P* < 0.05); ^#^significant difference from NH (*P* < 0.05). HH, continuous hypoxia; HIIE, high‐intensity interval exercise; HN, exercise in hypoxia; NH, recovery in hypoxia; NN, exercise under normoxia.

Heart rate responses during the work bouts displayed a main effect of condition (*P* < 0.001) and time (*P* < 0.001). Specifically, HR was higher in HN (all *P* < 0.001, *g* = 0.45–0.57; Figure 3a) compared to NN (5 [3–7] bpm), HH (5 [3–7] bpm) and NH (7 [4–9] bpm). Irrespective of conditions, HR was higher in intervals 3, 4, 5 and 6 when compared to interval 1 (all *P* < 0.001, *g* = 0.40–0.58). When expressed as a percentage of the HR_max_, the HR during the 1 min bouts (pooled average from intervals 1–6) were 85.8 ± 8.2%, 85.9 ± 7.4%, 85.4 ± 6.8% and 89.0 ± 6.0% for NN, HH, NH and HN, respectively.

Changes in $S_{pO_{2}}$ during the work bouts displayed a significant interaction effect (*P* = 0.014). Specifically, $S_{pO_{2}}$ was lower in HH compared to NN (all *P* < 0.001, *g* = 3.07–3.95) and NH (all *P* < 0.001, *g* = 1.19–3.49) across intervals 1–6, and lower than HN during interval 2 (*P* = 0.048, *g* = 0.64), 4 (*P* = 0.012, *g* = 0.62) and 5 (*P* = 0.002, *g* = 0.73). $S_{pO_{2}}$ was lower in HN compared to NN (all *P* < 0.001, *g* = 1.93–3.53) and NH (all *P* < 0.001, *g* = 0.66–3.20) across intervals 1–6. Change in $S_{pO_{2}}$ during the recovery bouts displayed a main effect of condition (*P* < 0.001). Compared with NN, $S_{pO_{2}}$ during recovery was significantly lower in HH (*P* < 0.001, *g* = 3.14) and NH (*P* < 0.001, *g* = 3.54). Additionally, $S_{pO_{2}}$ was significantly higher in HN compared to HH (*P* < 0.001, *g* = 2.60) and NH during recovery (*P* < 0.001, *g* = 2.80).

**FIGURE 4:**
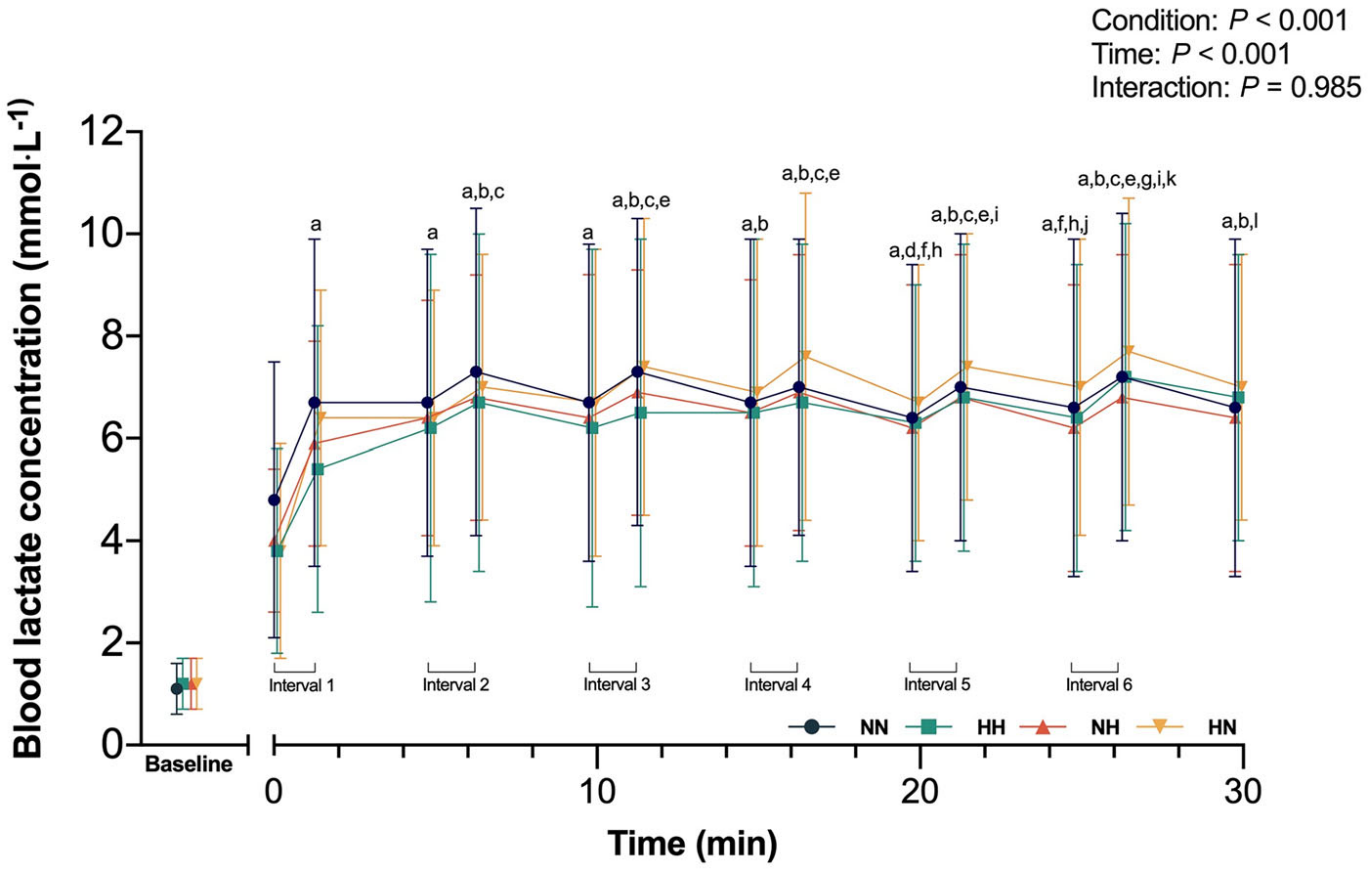
Blood lactate concentration measured at baseline (before any exercise was performed), 1 min before the start of exercise (0 min), and pre‐ (∼45 s before the start of the work bout) and post‐completion (∼15 s after the work bout) of each 1 min bout during the high intensity interval exercise under normoxic exercise–normoxic recovery (NN; blue), hypoxic exercise–hypoxic recovery (HH; green), normoxic exercise–hypoxic recovery (NH; orange), and hypoxic recovery–normoxic recovery (HN; yellow). ^a^Significant difference from 0 min (*P* < 0.05); ^b^significant difference from interval 1 (post‐exercise) (*P* < 0.05); ^c^significant difference from interval 2 (pre‐exercise) (*P* < 0.05); ^d^significant difference from interval 2 (post‐exercise) (*P* < 0.05); ^e^significant difference from interval 3 (pre‐exercise) (*P* < 0.05); ^f^significant difference from interval 3 (post‐exercise) (*P* < 0.05); ^g^significant difference from interval 4 (pre‐exercise) (*P* < 0.05); ^h^significant difference from interval 4 (post‐exercise) (*P* < 0.05); ^i^significant difference from interval 5 (pre‐exercise) (*P* < 0.05); ^j^significant difference from interval 5 (post‐exercise) (*P* < 0.05); ^k^significant difference from interval 6 (pre‐exercise) (*P* < 0.05); ^l^significant difference from interval 6 (post‐exercise) (*P* < 0.05). HH, continuous hypoxia; HN, exercise in hypoxia; NH, recovery in hypoxia; NN, exercise under normoxia.

Changes in blood lactate concentration displayed a main effect of condition (*P* < 0.001) and time (*P* < 0.001). Blood lactate concentration was higher in NN compared to HH (*P* = 0.003, *g* = 0.13; Figure 4) and NH (*P* = 0.01, *g* = 0.12). Additionally, blood lactate concentration in HN was higher than HH (*P* < 0.001, *g* = 0.17) and NH (*P* = 0.001, *g* = 0.16). When normalized by MPO, blood lactate concentration was higher in HH compared to NN (*P* = 0.02, *g* = 0.15) and NH (*P* < 0.001, *g* = 0.25), and higher in HN compared to NN (*P* = 0.013, *g* = 0.19) and NH (*P* < 0.001, *g* = 0.30).

### 3.3 Muscle oxygenation

Changes in TSI area above the curve displayed a main effect of condition (*P* < 0.001) and time (*P* < 0.001). Specifically, TSI area above the curve was greater in HN relative to NN (*P* < 0.001, *g* = 0.66), HH (*P* < 0.001, *g* = 0.59) and NH (*P* < 0.001, *g* = 0.58; Table 2). Additionally, changes in TSI above the curve were larger in HH compared to NN (*P* = 0.01, *g* = 0.12) and NH (*P* = 0.003, *g* = 0.06). Irrespective of condition, changes in TSI area above the curve were larger during intervals 2, 3, 4, 5 and 6 compared to interval 1 (all *P* < 0.001).

**TABLE 2:** Change in area above the curve of tissue saturation index (TSI) during high intensity interval exercise under normoxia (NN), continuous hypoxia (HH), recovery in hypoxia (NH) and exercise in hypoxia (HN).

	Interval 1	Interval 2	Interval 3	Interval 4	Interval 5	Interval 6	Condition	Time	Interaction
NN	610 ± 243	529 ± 221	495 ± 214	443 ± 202	443 ± 188	442 ± 192	*P* < 0.001	*P* < 0.001	*P* = 0.997
HH	637 ± 188^a^	523 ± 171^a^	492 ± 192^a^	467 ± 180^a^	472 ± 166^a^	517 ± 194^a^			
NH	678 ± 307^b^	519 ± 278^b^	471 ± 254^b^	449 ± 239^b^	430 ± 250^b^	473 ± 256^b^			
HN	910 ± 404^a,b,c^	738 ± 373^a,b,c^	670 ± 347^a,b,c^	656 ± 371^a,b,c^	606 ± 366^a,b,c^	642 ± 379^a,b,c^			

*Note*: Data are means ± standard deviation. Area above the curve of TSI (expressed in % 60/s) is measured from start to end of each 1 min work bout for intervals 1–6 during the high intensity interval exercise. ^a^Significant difference from NN. ^b^Significant difference from HH. ^c^Significant difference from NH.

### 3.4 Perceptual responses

**FIGURE 5:**
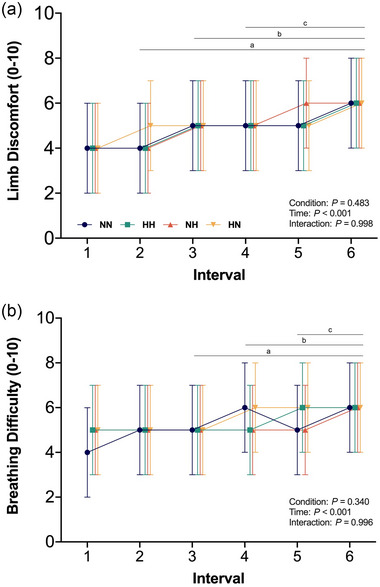
Ratings of limb discomfort (a) and breathing difficulty (b) during each interval of the HIIE under normoxic exercise–normoxic recovery (NN; blue), hypoxic exercise–hypoxic recovery (HH; green), normoxic exercise–hypoxic recovery (NH; orange), and hypoxic recovery–normoxic recovery (HN; yellow). ^a^Significant difference from interval 1 (*P* < 0.05); ^b^significant difference from interval 2 (*P* < 0.05); ^c^significant difference from interval 3 (*P* < 0.05). HH, continuous hypoxia; HIIE, high‐intensity interval exercise; HN, exercise in hypoxia; NH, recovery in hypoxia; NN, exercise under normoxia.

Limb discomfort (*P* = 0.48; Figure 5a) and breathing difficulty (*P* = 0.34, Figure 5b) did not differ between conditions. Limb discomfort (*P* ≤ 0.03) and breathing difficulty (*P* ≤ 0.04) increased across intervals, irrespective of condition.

## 4 DISCUSSION

This study examined the effects of intermittent hypoxia during perceptually regulated HIIE on exercise performance and physiological responses in inactive overweight or obese individuals. The results confirmed our initial hypothesis that intermittent hypoxic exposure, whether during the work or recovery, during HIIE helped mitigate the declines in mechanical output observed when exercise is performed in continuous hypoxia. Specifically, when hypoxia was implemented exclusively during recovery, changes in exercise performance closely resembled that in normoxia. However, contrary to our hypothesis, the addition of hypoxia, whether during the work or recovery phase, had minimal influence on internal loads, assessed by heart rate and blood lactate responses, when compared to HIIE in normoxia.

This study used a perceptually regulated HIIE protocol, where individuals were asked to maintain an RPE of 16 and 10 during work and recovery bouts, respectively. Participants were allowed to vary their work rate while adhering to the pre‐determined exertion targets. Given that participants were inactive, it is tenable to expect that their lack of exercise experience may result in large fluctuations in mechanical output and, consequently, exaggerated changes in physiological responses. In this study, mechanical output decreased across intervals, with the largest drop in mechanical output observed when HIIE was conducted in continuous hypoxia. Despite these changes in mechanical output, both physiological (e.g. HR) and perceptual responses were largely consistent during exercise across all conditions. This suggests that regardless of hypoxia manipulations, when unacclimatized individuals are required to exercise at an RPE of 16, they are able to self‐select their mechanical output so that exercise intensity does not exceed the pre‐determined level of perceptual stress. Consequently, the adjustments in mechanical output served to regulate physiological responses during exercise such that similar HR was achieved across conditions. These observations are consistent with previous findings (Brosnan et al., 2000) showing that elite female cyclists performing HIIE in hypoxia (3 × 10 min maximum work bouts in hypoxia interspersed with 5 min active recovery in normoxia) autoregulated their exercise intensity such that RPE and HR are comparable to HIIE in normoxia. Perceptually regulated exercise may therefore limit excessive perceptual stress which may contribute to exercise disengagement. This underscores the usefulness of RPE as a valuable tool when prescribing HIIE (in normoxia or hypoxia), in the inactive, overweight or obese populations.

This study manipulated hypoxia provision during HIIE such that hypoxia was implemented only during the work or recovery bouts to determine the extent to which declines in mechanical output associated with hypoxia can be attenuated. Compared to HIIE in normoxia, the decrease in performance was less pronounced when HIIE was performed in intermittent hypoxia rather than continuous hypoxia. In particular, mechanical output in normoxia (NN) was not different from the condition where hypoxia was applied solely during the recovery phase (NH). Interestingly, despite hypoxia being applied during recovery (NH), the self‐selected mechanical output during recovery was similar to the condition in normoxia (NN). Limited studies (Dellavechia de Carvalho et al., 2023; Papoti et al., 2023; Roels et al., 2005) have explored the application of hypoxia only during the recovery phase of a HIIE (i.e., work bouts are performed in normoxia). Of note, such a hypoxia protocol has largely been applied in HIIE where the workload during the work phase is fixed (e.g. 120% of peak running velocity achieved during a graded exercise test) (Dellavechia de Carvalho et al., 2023). Nonetheless, our results show that implementing hypoxia only during the recovery phase resulted in comparable performance levels (MPO) to HIIE in normoxia. However, it remains to be determined whether the acute metabolic stress is amplified using such a hypoxia protocol, and whether this would lead to more pronounced longer‐term adaptations than HIIE in normoxia.

When hypoxia was implemented only during the work phase, that is, recovery in normoxic conditions (HN), exercise performance was significantly higher than HH, but somewhat lower compared to NN. Unlike conventional high‐intensity exercise in hypoxia (Balsom et al., 1994), the use of normoxic recovery periods may have played a role to minimize cumulative fatigue during repeated high‐intensity bouts. This result aligns with previous research where all‐out running performance lasting up to 60 s is largely unaltered in hypoxia ($F_{iO_{2}}$ of 0.13) compared to normoxia (Weyand et al., 1999). That said, our results differ from a recent study in which normoxic recovery was introduced between sets of repeated sprints in hypoxia in female rugby seven players and did not mitigate declines in sprint performance (Brocherie et al., 2023). However, differences in exercise protocol (repeated maximal sprints vs. perceptually regulated interval exercise), severity of hypoxia ($F_{iO_{2}}$ of ∼0.10 vs. ∼0.14), and participants characteristics may contribute to these discrepancies.

Despite hypoxia manipulation during HIIE, HR responses were not different between conditions, except for higher HR in HN compared to NN, HH and NH (Figure 3a). The elevated HR in HN likely reflects a higher relative intensity and increased physiological strain. Globally, there was a gradual increase in reported values for limb discomfort and breathing difficulty, with values fluctuating between ∼4 and 6 from the first to last interval. However, perceptual responses including limb discomfort and breathing difficulty did not differ between conditions. These findings seem to reaffirm our finding that inactive overweight individuals who are not acclimatized to hypoxia are likely to adjust their workload resulting in relatively similar heart rate and/or perceptual responses.

The pattern of muscle oxygenation showed similar fluctuations throughout the HIIE in all conditions, primarily due to the repeated transitions from recovery to exercise. An apparent decrease in muscle oxygenation was observed during the 1 min bouts in all conditions (Figure 1). Accordingly, the decrease in muscle oxygenation during exercise was accentuated when HIIE was performed in HN. This could be explained by the oxygen cascade from blood to tissue (Richardson et al., 2006), where a decrease in $F_{iO_{2}}$ during the transition from recovery to exercise (in HN) and an increase in muscle oxygen consumption are likely to induce the greatest reduction in muscle oxygenation. Whilst similar patterns of fluctuations in muscle oxygenation during high intensity interval exercise have been reported (Combes et al., 2018), our findings suggest that the use of hypoxia only during the work bouts could possibly accentuate these changes. Importantly, these fluctuations in muscle oxygenation and metabolic rate during HIIE have been purported to be an important factor mediating longer‐term exercise adaptation (Combes et al., 2015; Daussin et al., 2008). However, whether greater fluctuations in muscle oxygenation during HIIE induced by hypoxia (implemented only during the work phase) may enhance longer‐term physiological adaptations associated with HIIE in normoxia requires further research.

Reduced oxygen availability may lead to increased anaerobic glycolysis (Gladden, 2004; Parolin et al., 2000) resulting in increased blood lactate levels. Therefore, when the absolute work rate during HIIE in hypoxia – whether implemented continuously or intermittently – matches that in normoxia (NN), it is reasonable to expect that the effects of hypoxia may contribute to elevated blood lactate levels. This expectation was supported by findings showing that blood lactate response during high‐intensity exercise in hypoxia is comparable to or even higher than that during high‐intensity exercise in normoxia, even when the absolute workload is reduced (Brosnan et al., 2000; Parolin et al., 2000). For instance, Brosnan et al. (2000) showed that repeated sprints in hypoxia ($F_{iO_{2}}$ ∼0.17; with recovery in normoxia between sets) led to reduced performance (∼5%) but similar lactate response compared to normoxia. Accordingly, we expected that HN would elicit the highest lactate response. However, despite exercise performance in HN being relatively comparable to NN (pooled average from set 1–6: ∼98% vs. ∼99% of *Ẇ*_peak_), and the presence of pronounced systemic hypoxaemia and localized deoxygenation, HIIE during HN did not elicit a higher lactate response compared to HIIE in normoxia. Nonetheless, when normalized to MPO, lactate response was significantly higher in HN and HH. This suggests that the effects of hypoxia enhanced blood lactate response during the HIIE.

Another interesting finding was that the lactate response in NH was lower compared to NN despite comparable exercise performance and the addition of hypoxia during recovery. This result differs from a previous study showing that lactate response was comparable under hypoxic (hypoxia implemented during recovery only) and normoxic conditions during HIIE (ten 1 min running efforts at 120% of ${\dot{V}}_{O_{2}\mathrm{peak}}$, interspersed with 2 min of passive recovery) (Dellavechia de Carvalho et al., 2023). However, this discrepancy could be attributed to the intensity of exercise, which in the previous study was fixed at 120% of ${\dot{V}}_{O_{2}\mathrm{peak}}$. Although speculative, it is possible that recovery in hypoxia may have impaired recovery, for example, slower phosphocreatine resynthesis (Haseler et al., 2007). In particular, decreases in anaerobic energy contribution may not necessarily result in a proportional decrease in power output (Bogdanis et al., 1998). This is because an increase in substrate availability, for example, free phosphate, may stimulate oxidative phosphorylation to maintain the same ATP turnover as in normoxia. However, we acknowledge that changes in blood lactate concentration primarily reflect lactate appearance, namely skeletal muscle lactate production and efflux into blood, and lactate disappearance by tissue uptake including skeletal muscle and liver. Therefore, this measurement may not fully elucidate the subtle changes in metabolic reactions during hypoxic exercise. Nevertheless, the results of this study indicate that hypoxia manipulation during HIIE does not elicit greater blood lactate level than in normoxia.

### 4.1 Limitations and additional considerations

As exercise in this study was perceptually regulated, it could be argued that the decrease in exercise performance in oxygen‐deprived conditions is due to pacing and/or the effects of hypoxia. Ideally, to exclude the possibility of pacing influencing our results, a control condition in normoxia, in which HIIE is performed at an identical absolute intensity as in continuous hypoxia, could have been included. However, evidence of pacing (Abbiss & Laursen, 2008), such as an ‘end spurt’ was not observed. Furthermore, the consistency in physiological and perceptual responses in this study suggests that inactive, overweight individuals who are unacclimatized to hypoxia can effectively regulate their exercise intensity to achieve the pre‐determined exertion level. Inter‐individual difference in exercise performance was evident in this study; this could in part be attributed to sex differences. For instance, we observed that absolute power output (e.g. *Ẇ*_peak_) was evidently higher in males than females. This could be due to underlying physiological difference (e.g. body composition) as well as sex‐specific differences in response to hypoxia (Raberin et al., 2024). In this context, the normalization of power output to body mass attenuated some of these differences (James et al., 2023). Whilst evidence has highlighted several plausible physiological mechanisms (e.g. fluctuations in sex hormones) that may explain difference in responses to hypoxia between sexes, their influence on exercise performance remains unclear (Raberin et al., 2024). It is important to note, however, that sex difference is one but not the only factor influencing responses to hypoxia and/or exercise performance. Another limitation is that changes in muscle oxygenation was examined from the gastrocnemius lateralis in this study. We acknowledge that the contractile activity of the gastrocnemius lateralis tends to be more variable than the quadriceps muscles, which are the primary muscles involved in cycling (Hug & Dorel, 2009). Nonetheless, we chose to examine muscle oxygenation in the gastrocnemius lateralis because adipose tissue thickness, which can affect NIRS measurements, is typically larger in the thigh compared to the calf (Möller et al., 2000).

### 4.2 Conclusion

This study showed that intermittent hypoxia, implemented during either the work or recovery phase of HIIE, attenuated significant declines in mechanical output associated with continuous hypoxia. However, hypoxia applied during the work phase resulted in increased physiological responses (HR) compared to HIIE in normoxia. Exercise performance comparable to that in normoxia can be achieved when hypoxia is implemented *exclusively* during the recovery phase of HIIE. Hypoxia applied only during recovery does not alter acute physiological or metabolic responses (blood lactate) beyond those in normoxia.

## AUTHOR CONTRIBUTIONS

Jacky Soo, Paul Goods, Olivier Girard, Louise Deldicque, Nathan Lawler, and Timothy Fairchild contributed to the conception and design of the study. Jacky Soo collected the data. Jacky Soo, Paul Goods, and Timothy Fairchild were responsible for the analysis and interpretation of data. Jacky Soo wrote the first draft of the manuscript. All authors have reviewed the manuscript and provided critical feedback. All authors have approved the final version of the manuscript and agree to be accountable for all aspects of the work in ensuring that questions related to the accuracy or integrity of any part of the work are appropriately investigated and resolved. All persons designated as authors qualify for authorship, and all those who qualify for authorship are listed.

## CONFLICT OF INTEREST

The authors declare no conflicts of interest.

## FUNDING INFORMATION

None.

## Data Availability

The data that support the findings of this study are available from the corresponding authors upon reasonable request.
